# Injury Rates Among Children With Autism Spectrum Disorder With or Without Attention-Deficit/Hyperactivity Disorder

**DOI:** 10.1001/jamanetworkopen.2024.59029

**Published:** 2025-02-10

**Authors:** Dorit Shmueli, Talish Razi, Moran Almog, Idan Menashe, Aviva Mimouni Bloch

**Affiliations:** 1 Child Development, Clalit Health Services, Tel Aviv, Israel; 2 Community Medical Services Division, Clalit Health Services, Tel Aviv, Israel; 3 Clalit Health Services, Tel Aviv, Israel; 4 Department of Epidemiology, Biostatistics and Community Health Sciences, Ben-Gurion University of the Negev, Beer-Sheva, Israel; 5 The Azrieli National Center for Autism and Neurodevelopment Research, Ben-Gurion University of the Negev, Beer-Sheva, Israel; 6 Child Development Center, Loewenstein Rehabilitation Medical Center, Raanana, Israel and Faculty of Medicine, Tel Aviv University, Tel Aviv, Israel

## Abstract

**Importance:**

Injuries are a major cause of morbidity and mortality among children with neurodevelopmental conditions. Identifying injuries associated with this vulnerable population could inform specific preventive actions.

**Objective:**

To compare the injury risk among children with autism spectrum disorder (ASD), attention-deficit/hyperactivity disorder (ADHD), or coexisting ASD and ADHD with children with typical development (TD).

**Design, Setting, and Participants:**

This population-based cohort study involved 325 412 children born between 2005 and 2009, followed up until the end of 2021. Data analysis was conducted from February 2 to November 14, 2023. The study data were extracted from the Clalit Health Service (CHS) database, which contains comprehensive clinical and sociodemographic data on all members of the CHS admitted to the emergency departments (EDs) of all hospitals in Israel during the study period. Children who were members of CHS born between 2005 and 2009 were classified into 4 groups: ASD, ADHD, ASD and ADHD, and TD (control).

**Main Outcomes and Measures:**

Negative binomial regression models were used to assess incidence rate ratios (IRRs) of ED visits between the study groups and the control group. These models were adjusted for birth year, sector, and socioeconomic status. IRRs for specific injuries were also assessed. The statistical significance of IRRs was assessed using 95% CIs.

**Results:**

A total of 325 412 children (163 183 boys [50%]) were included in the study. Children with ASD, ADHD, or both had significantly higher ED visit rates than children with TD (IRR, 1.48 [95% CI, 1.42-1.55], 1.45 [95% CI, 1.39-1.52], and 1.29 [95% CI, 1.28-1.30], respectively). Children with ADHD had also higher rates of ED visits due to physical injuries compared with controls (IRR, 1.18 [95% CI, 1.16-1.20]), whereas children with ASD with or without ADHD did not (IRR, 0.96 [95% CI, 0.89-1.05] and 0.91 [95% CI, 0.83-1.00], respectively). A focused analysis of the injury profiles revealed that children with ASD or comorbid ASD and ADHD had higher rates of ingestion and inhalation injuries (IRR, 1.57 [95% CI, 1.06-2.25] and 1.80 [95% CI, 1.28-2.48], respectively) and lower rates of orthopedic injuries (IRR, 0.78 [95% CI, 0.69-0.89] and 0.83 [95% CI, 0.74-0.93], respectively), and animal-inflicted injuries (IRR, 0.44 [95% CI, 0.22-0.79] and 0.92 [95% CI, 0.60-1.35], respectively) than controls.

**Conclusions and Relevance:**

In this large cohort study, different types of injury were associated with children with ASD and ADHD. The causes underlying these associations should be further investigated to develop effective approaches for injury reduction among these children.

## Key Points

**Question:**

Are children with autism spectrum disorder (ASD), with or without attention-deficit/hyperactivity disorder (ADHD), at increased risk for physical injuries?

**Findings:**

In this population-based cohort study of 325 412 children, we found that children with ASD, irrespective of comorbid ADHD, had a similar emergency department visit rate due to injuries compared with typically developing children and a lower rate than that of children with ADHD without ASD. Furthermore, children with ASD without ADHD had lower rates of animal-inflicted and orthopedic injuries than typically developing children.

**Meaning:**

These findings suggest that children with ASD have a lower risk for certain injuries than children with ADHD.

## Teaser

This population-based cohort study compares the rates of injury among children in Israel with autism spectrum disorder (ASD) with or without attention-deficit/hyperactivity disorder (ADHD).

## Introduction

Injuries are a major cause of morbidity and mortality among children and adolescents.^1^ It is thus crucial to identify individuals or groups within this population who are at a high risk of sustaining substantial injuries. Such an effort will not only enhance our understanding of potential risks and protective measures but will also facilitate the development of strategies to mitigate these risks.

Two groups that are likely to be at heightened risk are children and adolescents with attention-deficit/hyperactivity disorder (ADHD) and those with autism spectrum disorder (ASD), the most prevalent developmental disabilities (with a prevalence of 11.4% and 2.8% for ADHD and ASD, respectively).^2,3^ Previous research has demonstrated that children with ADHD are more susceptible to injuries^4,5^ and have a higher rate of emergency department (ED) visits than children without ADHD.^6,7^ For example, Bonander et al^8^ found an increased risk of injury among school children with ADHD but not for those with ASD. These findings are often attributed to the impulsivity and lack of attention characteristic of children with ADHD. Similarly, the characteristics of children with ASD, which include difficulties with social interaction, sensitivity to sensory input, and a lack of flexibility, could, hypothetically, make children with ASD more prone to accidents. However, existing studies about children with ASD have yielded mixed results, with some studies suggesting an increased risk of injury compared with children with typical development (TD)^9,10^ and others indicating a decreased risk for youths with ASD vs youths without ASD or intellectual disability.^11^ Some studies have also suggested that individuals with ASD are at risk for suffocation, asphyxiation, drowning,^12^ poisoning,^13^ and hip fractures.^14^

Approximately 40% to 70% of children with ASD also have ADHD.^15,16,17^ It could be assumed that children with this common comorbidity would be at a higher risk for injury than children with each of these diagnoses alone. However, the data available on this issue are scarce. To the best of our knowledge, only 1 study has explored the risk of injury for individuals with both ASD and ADHD.^18^ That study found that whereas ADHD was a risk factor for significant injury, individuals with both ASD and ADHD were less vulnerable to injury.

In the present study, we addressed this issue of risk of injury in a cohort study of children with ASD, ADHD, or ASD and ADHD comorbidity. We compared the risk of injury between children with these diagnoses and identified the specific types of injury associated with each of these neurodevelopmental groups.

## Methods

### Study Population

In this cohort study, we followed a cohort of children born between January 1, 2005, and December 31, 2009, from birth until December 31, 2021. The study included only children who were members of the Clalit Health Service (CHS), the largest health management organization (HMO) in Israel, covering approximately 52% of the Israeli population. The standard practice of the CHS is to refer children suspected of having neurodevelopmental disorders to a child neurologist or psychiatrist for further evaluation. Diagnoses of ASD, ADHD, or other neurodevelopmental conditions are made according to *Diagnostic and Statistical Manual of Mental Disorders* (Fifth Edition) (*DSM-5*) criteria.^19^ This study was approved by the institutional review board of the Loewenstein Rehabilitation Medical Center. Obtaining informed consent from study participants was waived by the institutional review board given the retrospective nature of the study and the anonymity of the data. This study followed the Strengthening the Reporting of Observational Studies in Epidemiology (STROBE) reporting guideline.

### Data Extraction

The study data were extracted from the CHS database, which contains comprehensive clinical information, including chronic conditions, hospitalizations, ED visits, and medications. Sociodemographic data, such as age, sex, population sector (general Jewish population, Arab population, or ultraorthodox Jewish population), and socioeconomic status score (ranging from 1 [lowest] to 10 [highest]) were extracted for each enrolled patient. Children whose CHS membership had been discontinued, who had moved away from Israel, or who had died during the study period were excluded. We also excluded children who were referred to CHS clinics with a suspicion of abnormal development but were not diagnosed with either ASD or ADHD. Information about pediatric ED visits was extracted from the CHS hospital database and included the entire population, regardless of a diagnosis of ASD and/or ADHD.

### Study Group Ascertainment

In the CHS, a diagnosis of ASD is typically recorded in a child’s medical records, as a formal ASD diagnosis is required to access special services from government sources. In contrast, a formal diagnosis of ADHD does not confer any special benefits on the child and is therefore not systematically documented. For this reason, we defined children with ADHD as children with a formal ADHD diagnosis or children for whom stimulants had been purchased at least twice between 2005 and 2021.^20^ For the purpose of this study, 4 groups of children were defined: children with ASD without ADHD, children with ASD and ADHD, children with ADHD without ASD, and children with TD. All children who were members of CHS with no diagnosis of ADHD or ASD were included in the TD group, which served as the control group for the study (Figure).

**Figure.**
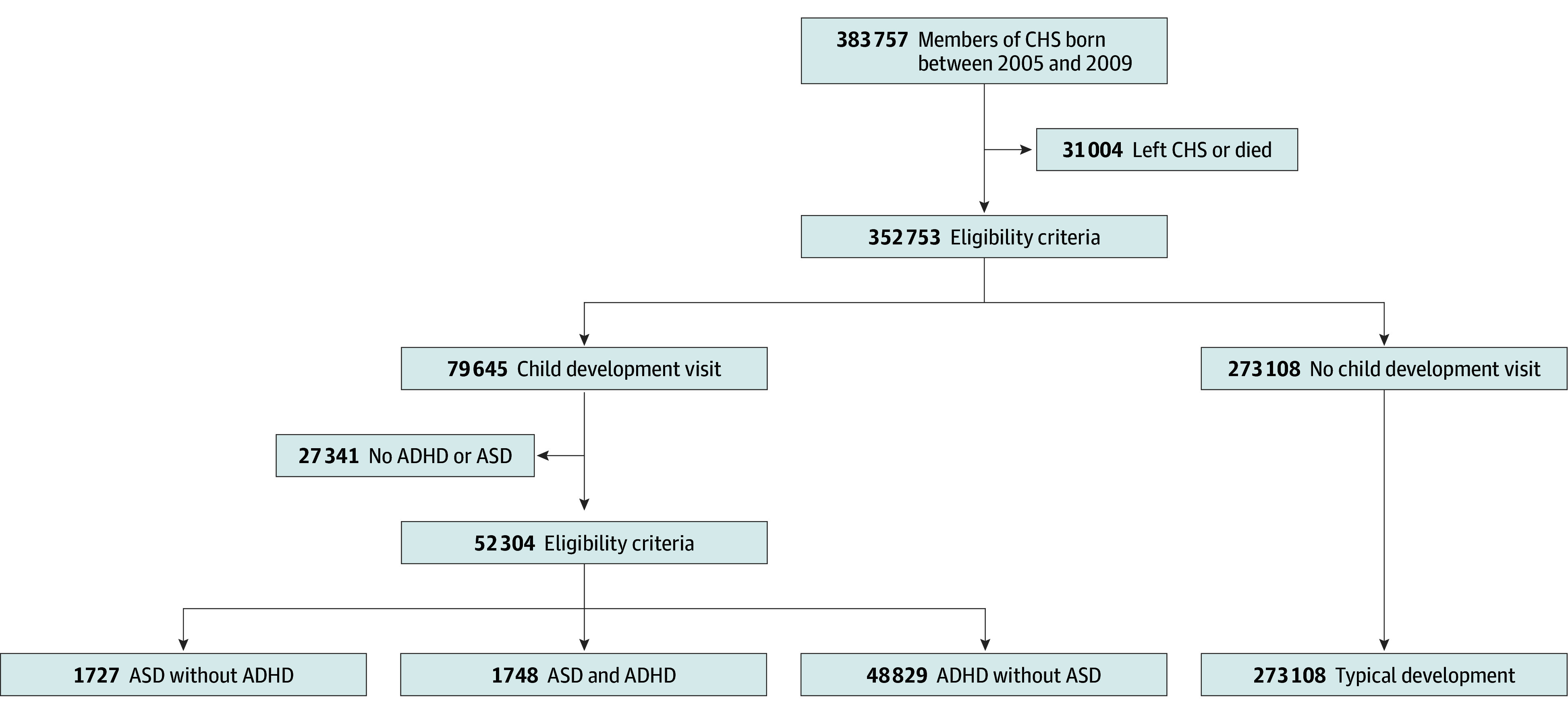
Flowchart of the Study Groups Ascertainment ADHD indicates attention-deficit/hyperactivity disorder; ASD, autism spectrum disorder; and CHS, Clalit Health Service.

### ED Visits and Injury Classification

CHS members are eligible for treatment in the EDs of any of 34 hospitals throughout Israel; of these, 9 are owned by the CHS, and 25 are public hospitals.^16^ Data on injury diagnoses, as recorded by an ED physician, were available to us only for ED visits to CHS hospitals. We used these diagnoses to classify the ED visits into injuries and noninjuries and to further classify the injuries into 9 predefined categories (ie, orthopedic injury; head, face, and neck injury; ingestions and inhalations; animal-inflicted injury; falls, not otherwise specified [NOS]; traffic accidents; burns; trunk injuries; and other injuries, as detailed in eTable in Supplement 1). Importantly, ED visits due to mental health conditions are usually referred to psychiatric EDs and were therefore not included in our data.

### Statistical Analysis

Negative binomial regression models were used to assess differences in incidence rate ratios (IRRs) of ED visits and injury types among the study group. These models were adjusted for sex, year of birth, sector, and socioeconomic status. The statistical significance of each IRR was assessed using a 95% CI. The statistical analysis was performed from February 2 to November 14, 2023, using R statistical software, version 4.0.1 (R Project for Statistical Computing). All *P* values were from 2-sided tests, and results were deemed statistically significant at *P* < .05

## Results

The study included a total of 325 412 children (163 183 boys [50%]); of these, 1727 (0.5%) were classified as having ASD without ADHD, 1748 (0.5%) as having ASD and ADHD, and 48 829 (15%) as having ADHD without ASD. Notably, the proportion of children with ASD (with or without ADHD) increased with birth year (from 18% of children with ASD and ADHD [315 of 1748] and 16% of children with ASD without ADHD [275 of 1727] born in 2005 to 23% of children with ASD and ADHD [409 of 1748] and 28% of children with ASD without ADHD [484 of 1727] born in 2009) (Table 1). Conversely, the proportion of children with ADHD without ASD decreased slightly across birth years (from 21% [10 205 of 48 829] to 18% [8906 of 48 829] for children born in 2005 and 2009, respectively). As expected, the male to female ratio was approximately 4:1 in both ASD groups and approximately 2:1 in the group of children with ADHD without ASD.

**Table 1.** Patient Characteristics in the Study Cohort

Characteristic	Overall (N = 325 412)	ASD without ADHD (n = 1727)	ASD + ADHD (n = 1748)	ADHD without ASD (n = 48 829)	TD (n = 273 108)
Year of birth, No. (%)					
2005	64 491 (20)	275 (16)	315 (18)	10 205 (21)	53 696 (20)
2006	65 958 (20)	298 (17)	309 (18)	10 283 (21)	55 068 (20)
2007	65 032 (20)	300 (17)	351 (20)	9662 (20)	54 719 (20)
2008	64 946 (20)	370 (21)	364 (21)	9773 (20)	54 439 (20)
2009	64 985 (20)	484 (28)	409 (23)	8906 (18)	55 186 (20)
Sex, No. (%)					
Female	162 229 (50)	402 (23)	310 (18)	16 225 (23)	145 292 (53)
Male	163 183 (50)	1325 (77)	1438 (82)	32 604 (67)	127 816 (47)
Sector, No. (%)					
General	185 563 (57)	1349 (78)	1484 (85)	38 962 (80)	143 768 (53)
Arab	115 605 (36)	266 (15)	158 (9)	5084 (10)	110 097 (40)
Ultraorthodox	24 244 (7)	112 (6)	106 (6)	4783 (10)	19 243 (7)
Socioeconomic score, median (IQR)	5 (3-7)	6 (4-7)	6 (5-8)	6 (4-7)	5 (3-7)

Abbreviations: ADHD, attention-deficit/hyperactivity disorder; ASD, autism spectrum disorder; TD, typical development.

Additionally, the proportion of Arab children among children with ASD, ADHD, or ASD and ADHD was remarkably lower in the Arab sector (266 of 1727 children with ASD [15%]; 5084 of 48 829 children with ADHD [10%]; and 158 of 1748 children with ASD and ADHD [9%]) than their proportion in the general population (115 605 of 325 412 [36%]). Last, the socioeconomic scores for families of children with ASD (6 [IQR, 4-7]), ADHD (6 [IQR, 4-7]), and ASD and ADHD (6 [IQR, 5-8]) were slightly higher than the population median (5 [IQR, 3-7]) (Table 1). Notably, during the period of data analysis, there was a decrease in the proportion of children with ADHD (from 16% of children born in 2005 [10 520 of 64 491] to 14% of children born in 2009 [9315 of 64 985]) and an increase in the proportion of children with ASD (from 0.9% [590 of 64 491] to 1.4% [893 of 64 985]).

During the study period, a total of 1 072 980 ED visits were recorded: 565 594 visits to CHS hospitals and 507 386 visits to other hospitals (Table 2). No notable differences were observed between these 2 groups, except for a slightly lower rate of visits of children from the Arab sector to CHS hospitals than to other hospitals (29% [164 707 of 565 594] vs 37% [188 948 of 507 386]).

**Table 2.** Characteristics of Emergency Department Visits, Stratified by CHS or Other Hospitals

Characteristic	Overall (N = 1 072 980)	Other (n = 565 594)	CHS (n = 507 386)
Type, No. (%)			
ASD without ADHD	8561 (0.8)	4522 (0.8)	4039 (0.8)
ASD + ADHD	8696 (0.8)	4935 (0.9)	3761 (0.7)
ADHD without ASD	210 908 (20)	114 293 (20)	96 615 (19)
Typical development	844 815 (79)	441 844 (78)	402 971 (79)
Year of birth, No. (%)			
2005	233 851 (22)	125 455 (22)	108 396 (21)
2006	226 730 (21)	121 054 (21)	105 676 (21)
2007	215 887 (20)	114 506 (20)	101 381 (20)
2008	203 278 (19)	105 650 (19)	97 628 (19)
2009	193 234 (18)	98 929 (17)	94 305 (19)
Sex, No. (%)			
Female	457 262 (43)	243 588 (43)	213 674 (42)
Male	615 718 (57)	322 006 (57)	293 712 (58)
Sector, No. (%)^a^			
General Jewish	667 056 (62)	372 879 (66)	294 177 (58)
Arab	353 655 (33)	164 707 (29)	188 948 (37)
Ultraorthodox	52 269 (4.9)	28 008 (5.0)	24 261 (4.8)
Socioeconomic score, median (IQR)	5 (3-7)	6 (4-7)	5 (3-6)

Abbreviations: ADHD, attention-deficit/hyperactivity disorder; ASD, autism spectrum disorder; CHS, Clalit Health Service.

^a^ Sector is based on the patient’s clinic location obtained from the medical records.

IRRs of ED visits and injuries for children with ASD and/or ADHD (compared with children with TD) are presented in Table 3. Children with ASD, ADHD, or both had significantly higher rates of ED visits than children with TD (IRR, 1.48 [95% CI, 1.42-1.55] for children with ASD without ADHD; IRR, 1.45 [95% CI, 1.39-1.52] for children with ASD and ADHD; IRR, 1.29 [95% CI, 1.28-1.30] for children ADHD without ASD). A similar trend was observed for ED visits to CHS hospitals (Table 3). In CHS hospitals, 152 557 (30%) of the ED visits were related to injuries. Rates of injury were significantly higher for children with ADHD without ASD than for children with TD (IRR, 1.18 [95% CI, 1.16-1.20]), whereas rates for children with ASD (with or without ADHD) were slightly lower than those for children with TD, but the latter differences were not statistically significant (IRR, 0.96 [95% CI, 0.89-1.05] and 0.91 [95% CI, 0.83-1.00], respectively). The higher rates of ED visits among children with ADHD without ASD was seen across all types of injury, with the highest IRR being observed for ingestion and inhalation insults (IRR, 1.41 [95% CI, 1.29-1.54]). Interestingly, the IRR for inhalation and ingestion injuries was even higher for children with ASD with ADHD (IRR, 1.80 [95% CI, 1.28-2.48]) or without ADHD (IRR, 1.57 [95% CI, 1.06-2.25]). In contrast, children with ASD had lower rates of ED visits due to orthopedic injuries (with ADHD: IRR, 0.83 [95% CI, 0.74-0.93]; without ADHD: IRR, 0.78 [95% CI, 0.69-0.89]). Children with ASD without ADHD had half of the ED visit rates due to animal-inflicted injuries compared with children with TD (IRR, 0.44 [95% CI, 0.22-0.79]), whereas no such difference was seen for children with both ASD and ADHD.

**Table 3.** Incidence Rate Ratios of ED Visits and Injuries in Children With ASD and/or ADHD^a^

Variable	Events, No.	Injuries, %	Incidence rate ratio (95% CI)^b^		
			ASD without ADHD	ASD + ADHD	ADHD without ASD
Total ED visits	1 072 980	NA	1.48 (1.42-1.55)	1.45 (1.39-1.52)	1.29 (1.28-1.30)
ED visits (CHS)	507 386	NA	1.44 (1.33-1.56)	1.50 (1.39-1.63)	1.29 (1.26-1.31)
Total injuries (CHS)	152 557	30^c^	0.91 (0.83-1.00)	0.96 (0.89-1.05)	1.18 (1.16-1.20)
Orthopedic injuries	82 836	54^d^	0.78 (0.69-0.89)	0.83 (0.74-0.93)	1.16 (1.13-1.19)
Head, face, and neck injuries	50 804	33^d^	1.07 (0.94-1.20)	1.09 (0.98-1.21)	1.18 (1.15-1.21)
Ingestion and inhalation	4065	3^d^	1.57 (1.06-2.25)	1.80 (1.28-2.48)	1.41 (1.29-1.54)
Animal-inflicted injuries	3841	3^d^	0.44 (0.22-0.79)	0.92 (0.60-1.35)	1.20 (1.10-1.31)
Falls^e^	2931	2^d^	0.85 (0.46-1.42)	1.07 (0.65-1.66)	1.18 (1.07-1.32)
Traffic injury	2841	2^d^	1.22 (0.72-1.91)	0.63 (0.32-1.12)	1.34 (1.20-1.48)
Burns	2666	2^d^	1.61 (0.96-2.52)	1.47 (0.89-2.27)	1.20 (1.07-1.35)
Trunk injuries	2207	1^d^	0.66 (0.30-1.25)	0.83 (0.44-1.41)	1.32 (1.18-1.47)
Other	366	0^d^	0.57 (0.03-2.61)	1.88 (0.57-4.66)	1.54 (1.16-2.03)

Abbreviations: ADHD, attention-deficit/hyperactivity disorder; ASD, autism spectrum disorder; CHS, Clalit Health Service; ED, emergency department; NA, not applicable.

^a^ Study population (N = 306 130); children visiting the ED at CHS hospitals (n = 145 671).

^b^ Adjusted for year of birth, sector, sex, and socioeconomic status.

^c^ Percentage of total ED admissions at CHS hospitals.

^d^ Percentage of total injuries seen at CHS hospitals.

^e^ Not otherwise specified.

## Discussion

This study—the most comprehensive to date, to our knowledge—investigated, among other things, the rate of ED visits and injury rates among children with ASD, ADHD, or both ASD and ADHD compared with children with TD. Our findings indicate that children with these diagnoses had higher rates of ED visits for all medical reasons compared with children with TD. These findings are in line with previously reported overall greater emergency medical use for children with neurodevelopmental disorders.^21,22^ However, the reasons for visits related to injuries varied between among groups. Compared with children with TD, children with ADHD alone had higher rates of ED visits due to all types of injury, whereas children with ASD alone had higher rates of ED visits due to inhalation and ingestion injuries and lower rates due to orthopedic or animal-inflicted injuries. These findings underscore the distinct profiles for ED visits for children with ASD and/or ADHD.^21^

We found that children with ADHD had higher injury rates than children with TD, but the total injury rates for children with ASD were similar to those of children with TD, regardless of ADHD comorbidity. This finding suggests that ASD was the dominant diagnosis in the context of ED visits due to injury. This conclusion aligns with studies conducted in Sweden and the US; the former found fewer injuries among children with ASD than among children with ADHD,^8^ and the latter reported that comorbid ASD decreased the risk of injury for individuals with ADHD.^18^ The notion that the characteristics of ASD may protect individuals from physical injuries is intriguing. For example, many children with ASD exhibit significant levels of comorbid anxiety, which tends to deter them from engaging in various physical activities, thereby reducing their exposure to physical injuries.^23^ Another explanation for this notion may be related to the closer supervision of children with ASD by adults,^24^ including immediate caregivers, educators, and therapists.^25,26,27^ In Israel, a child with ASD is entitled to up to 30 hours a week of assistance or therapy in the educational system at no cost.^28^ This level of support is not available to most children with ADHD (only children with severe ADHD are entitled to extra support^28^) or to children with TD. Additionally, families of children with ASD receive parking and other accessibility benefits that may further reduce the risk of injury for these children.

Our study also revealed different injury profiles for children with ASD vs those with ADHD. Specifically, when compared with children with TD, children with ADHD had a higher IRR for all injuries, whereas children with ASD had a noticeably lower IRR for orthopedic and animal-inflicted injuries and a much higher IRR for ingestion and inhalation injuries. A possible explanation for the differences between the 2 groups in the IRR of ingestion and inhalation injuries is that such injuries typically occur at home, where the differences in supervision between ADHD and ASD groups are likely to be less marked. An alternative potential explanation is that ingestion is a common method of attempted suicide, a finding that is in accordance with more prevalent psychiatric problems among patients with ASD.^6^ Unfortunately, we did not have details regarding the reason for the ingestion injuries and, therefore, could not investigate whether they were deliberate or accidental.

An incidental finding from our data suggested that during the period of data analysis, there was a decrease in the proportion of children with ADHD (from 16% of children born 2005 to 14% of children born in 2009) and an increase in the proportion of children with ASD (from 0.9% to 1.4%). These contrasting trends might be because of a shift in diagnoses, with children previously diagnosed as having ADHD being currently diagnosed as having ASD. The overlap of ASD and ADHD symptoms has indeed been widely discussed in recent years,^29,30,31^ and its effect on diagnosis trends is a topic worthy of further research.

### Limitations

The results of this study should be considered with the following limitations. First, data on injuries were available only for patients visiting CHS hospitals, which accounted for approximately half of the cohort. However, the characteristics of patients visiting the CHS hospitals did not differ significantly from those of the other patients, apart from a lower representation of the Arab population. Thus, our findings about the injuries could be generalized to the entire study population. Second, we did not have information regarding the reasons for the injuries in our dataset. Furthermore, information on the behavioral and emotional difficulties associated with the ED visits was not consistently recorded in the CHS medical files, preventing the inclusion of these difficulties in the analysis. In addition, data regarding family characteristics and lifestyle that could also contribute to children’s injuries and ED visits were not available to us. Thus, caution should be applied when drawing conclusions regarding the possible reasons leading to the differences in ED visits and injury profiles among the groups in our study.

## Conclusions

This cohort study found differences in injury rates between children with ASD and those with ADHD that may be associated with the closer daily supervision provided for children with ASD, but not for children with ADHD, in Israel by law. Thus, further studies should focus on evaluating the effects of adult supervision on the rates of injuries among children with ASD, ADHD, or both ASD and ADHD and examine possible injury prevention programs that can highlight the type of support that is most beneficial for these children.

## Notes

Supplement 1. **eTable.** Classification of Injuries Into Nine Predefined Categories

Supplement 2.
Data Sharing Statement
