# Supplementary material for: Injury Rates Among Children With Autism Spectrum Disorder With or Without Attention-Deficit/Hyperactivity Disorder
Source: JAMA Netw Open. 2025 Feb 10;8(2):e2459029. doi: 10.1001/jamanetworkopen.2024.59029 (PMC11811789; doi:10.1001/jamanetworkopen.2024.59029)
Supplement: Supplement 2. — Data Sharing Statement [file jamanetwopen-e2459029-s002.pdf]

Data Sharing Statement

Shmueli. Injury Rates Among Children With Autism Spectrum Disorder With or Without Attention-Deficit/Hyperactivity Disorder. *JAMA Netw Open*. Published February 10, 2025. doi:10.1001/jamanetworkopen.2024.59029

Data
Data available: No

Additional Information
Explanation for why data not available: Data summary that supports the findings of this study is available on request from the corresponding author, [IM]. The data are not publicly available due to [restrictions e.g. their containing information that could compromise the privacy of research participants].
